# Medical Students’ Perceptions on Identifying and Addressing Emotional Responses in Emergency Medicine: Pilot Investigation

**DOI:** 10.2196/50827

**Published:** 2024-01-10

**Authors:** Anish Kumar Agarwal, Rachel Gonzales, Cory Munden, DaCarla Albright, Suzana Tsao

**Affiliations:** 1 Department of Emergency Medicine University of Pennsylvania Philadelphia, PA United States; 2 Center for Health Care Transformation and Innovation Penn Medicine Philadelphia, PA United States; 3 Department of Obstetrics and Gynecology University of Pennyslvania Philadelphia, PA United States

**Keywords:** well-being, burnout, medical education, coping, student, students, university, college, acute care, trauma, traumatic, emotion, emotional, stress, distress, psychological, cross-sectional, survey, surveys, critical, critically, perception, perspectives, prepared, preparedness

## Abstract

**Background:**

Training in acute care, such as emergency medicine (EM), where exposure to critically ill and injured patients is high, impacts the well-being of trainees and contributes to burnout. Investigating how, and if, trainees prepare for these situations is necessary to ensure they are supported.

**Objective:**

This study aimed to evaluate medical students’ perspectives and emotional preparedness for handling acute care and trauma.

**Methods:**

We conducted a pilot investigation using a remote digital survey of medical students during their EM clerkship at a large, urban academic institution. The primary outcome of interest was student-reported preparedness and comfort in handling trauma and critical care patient encounters. Secondary outcomes included awareness of well-being resources and comfort in accessing digital well-being resources.

**Results:**

A total of 57 medical students completed the voluntary digital survey, and half of the students (n=28, 49%) reported having witnessed the care of a critically ill or a penetrating trauma patient (eg, a victim of gun violence). A majority (n=40, 70%) had thought about how these events may impact them, and over half felt unprepared to identify the emotional impact these cases may have on them (n=31, 54%) or address the emotional or mental health impact (n=36, 63%). Less than a quarter (n=14, 25%) were aware of digital mental health resources, and 58% (n=33) did not feel fully comfortable connecting with resources if needed. Students who had previously witnessed critical care were significantly more likely to report feeling well prepared in identifying the emotional impact and addressing this impact.

**Conclusions:**

In this cross-sectional survey, students did not feel fully prepared to identify or address the emotional impact of working in EM. Additionally, they lacked awareness of or comfort with accessing digital institutional resources meant to support their well-being, such as a large web-based platform. These findings can help inform and guide interventions by educational and academic leaders. The aim would be to create and promote environments that empower students with tools to identify their own emotions and connect to well-being resources.

## Introduction

Health care–associated burnout persists in medicine and can be identified early in medical training [1-4]. This syndrome has negative impacts at the individual level, and it also affects patient care and the health care system by contributing to more medical errors, lower patient satisfaction, reduced productivity, poor clinical teaching and role modeling for trainees, increased cost, and increased physician attrition and ultimately contributes to physician suicide [5-8]. Physicians have a higher risk for suicide than the general population, and these mental health risks have been labeled as known “occupational hazards” [9,10]. Notably, the rate of US physicians annually completing suicide is estimated to be equivalent to the number of students in 3 graduating medical school classes [11,12].

Studies have demonstrated that the impacts of burnout and mental health symptoms, such as depression and anxiety, begin early in medical education and are found in medical students, residents, and physicians in training [2,13-15]. Studies have also found that rates of depression are higher for medical students than other trainees, suggesting this group may be particularly in need of interventions to support well-being and prevent burnout [11,14]. The strain and emotional toll of working in health care emerges early in training. For students, investigating how one identifies, processes, and copes with feelings of anxiety, sadness, depression, or stress related to their clinical experiences within clerkships remains understudied [16]. Emergency medicine (EM) physicians consistently report some of the highest rates of burnout, with EM often being referred to as the “center” of burnout [6,17]. EM physicians are 3 times more likely to be burned out compared to non-EM physicians [4].

Investing in strategies to help trainees identify their own emotions related to providing care, how to cope, and how to sustain well-being is critical for the future of the workforce [18]. Rather than awaiting burnout to evolve, examining how prepared these students feel is necessary to ensure that health systems and medical schools adequately support and proactively maintain medical students’ well-being. This is especially important given the rise of mental health symptoms and burnout in health care within the backdrop of the COVID-19 pandemic and social unrest related to racial injustice and rising gun violence.

The goal of this study was to assess medical students’ preparation during their EM rotation to understand how students self-identify their capacity to deal with emotionally charged clinical settings (eg, critical care cases or trauma).

## Methods

### Ethical Considerations

This pilot investigation used an electronic voluntary survey administered to second- and third-year medical students at the University of Pennsylvania in Philadelphia during their EM clerkship at a large, urban academic institution. The study was approved by the University of Pennsylvania Institutional Review Board (849318). All research methods, consent, and activities were performed in accordance with the university guidelines and regulations.

### Eligible Participants and Study Type

Inclusion criteria consisted of students in their second (preclerkship or M2) or third (clerkship or M3) year of medical school. This study was cross-sectional, and a voluntary response sample was used. There were no specific exclusion criteria, as other students were not invited to the survey.

### Recruitment Procedures

Participants were invited via email, completed informed consent, and were not compensated for participating. Data were collected and aggregated for analysis. The students at this medical school are routinely surveyed, and questions from this study were incorporated into the preexisting and ongoing school surveying. In total, the survey was sent to 161 students in their preclerckship (M2) and 153 students in their clerckship (M3) during the final week of classes in December 2021.

### Approach and Analysis

This cross-sectional pilot study was developed by the research team, with expertise in medical education (authors ST and DA), qualitative methods (AKA and RG), and clinician well-being (AKA). No previous instrument, to the knowledge of the study team, exists; thus, the instrument was developed and pilot-tested in this study. All answers were anonymous, and no demographic information was collected. The primary outcome of interest was student-reported preparedness and comfort in handling trauma and critical care patient encounters. Secondary outcomes included awareness of well-being resources available to them during their clerkship, feelings of preparedness, and comfort in accessing well-being resources. Comparisons were done using chi-square tests in Stata IC 16.1 (StataCorp), and a *P*<.05 was considered statistically significant.

## Results

A total of 57 medical students completed the voluntary survey; 26 (46%) of them were M2 students, and 31 (54%) were M3 students. Almost half (n=28, 49%) of the students reported having witnessed the care of a critically ill or injured patient (defined as a victim of gun violence). Most (n=40, 70%) students had thought about how these events may impact them, but most did not feel fully prepared to identify the emotional impact these cases may have on them (n=31, 55%) or prepared to address this emotional or mental health impact (n=36, 63%). Although resources are widely available to support students’ well-being at this institution, only 25% (n=14) were aware of these institutional resources to help them cope with the emotions involved with care, and 58% (n=33) did not feel fully comfortable connecting with resources if needed (Table 1).

Differences were identified between those students who had witnessed the care of a critically ill or injured patient and those who had not (Table 2). Students who had witnessed such care were more likely to feel well prepared in identifying the emotional impact of these cases (n=7, 25% vs n=0, 0%; *P*=.007) and in addressing this impact (n=9, 32% vs n=0, 0%; *P*=.001). No significant differences were found in student awareness of resources and their comfort in connecting with these resources to cope with the emotions involved with care.

**Table 1:** Medical students’ perspectives on the emotional impact and preparedness of caring for critical patients.

Questions			Values (N=57)
**Have you witnessed the immediate care of a critically ill or injured patient?**			
	Yes	28 (49)	
	No	29 (51)	
**Have you thought about how these events may impact you?**			
	Yes	40 (70)	
	No	15 (26)	
	N/A^a^	2 (4)	
**How prepared do you feel in identifying the emotional impact these cases may have?**			
	Well prepared	7 (12)	
	Prepared	18 (32)	
	Somewhat prepared	23 (40)	
	Not prepared	8 (14)	
	N/A	1 (2)	
**How prepared do you feel in addressing with the emotional impact these cases may have?**			
	Well prepared	9 (16)	
	Prepared	11 (19)	
	Somewhat prepared	25 (44)	
	Not prepared	11 (19)	
	N/A	1 (2)	
**Are you aware of university resources to support learners as they cope with the emotions involved with care?**			
	Yes	14 (25)	
	No	42 (74)	
	N/A	1 (2)	
**How comfortable you would feel in connecting with resources if needed?**			
	Very comfortable	7 (12)	
	Comfortable	16 (28)	
	Somewhat comfortable	23 (40)	
	Not comfortable	10 (18)	
	N/A	1 (1.8)	

^a^N/A: not applicable.

**Table 2:** Medical students’ perspectives on personal emotional reaction, preparation, and coping skills based on prior exposure.

Questions		Ever witnessed the care of a critically ill or injured patient (N=57)			
		Yes (n=28)	No (n=29)	*P* value	
**Have you thought about how these events may impact you?**					.32
	Yes	22 (78.6)	18 (62)		
	No	6 (21.4)	9 (31)		
	N/A^a^	0 (0.0)	2 (7)		
**How prepared do you feel in identifying the emotional impact these cases may have?**					.007
	Well prepared	7 (25.0)	0 (0)		
	Prepared	10 (35.7)	8 (28)		
	Somewhat prepared	10 (35.7)	13 (45)		
	Not prepared	1 (3.6)	7 (24)		
	N/A	0 (0.0)	1 (3)		
**How prepared do you feel in addressing with the emotional impact these cases may have?**					.001
	Well prepared	9 (32.1)	0 (0)		
	Prepared	6 (21.4)	5 (17)		
	Somewhat prepared	12 (42.9)	13 (45)		
	Not prepared	1 (3.6)	10 (35)		
	N/A	0 (0.0)	1 (4)		
**Are you aware of university resources to support learners as they cope with the emotions involved with care?**					.22
	Yes	9 (32.1)	5 (17)		
	No	19 (67.9)	23 (79)		
	N/A	0 (0.0)	1 (4)		
**How comfortable you would feel in connecting with resources if needed?**					.63
	Very comfortable	5 (17.9)	2 (7)		
	Comfortable	8 (28.5)	8 (28)		
	Somewhat comfortable	11 (39.3)	12 (41)		
	Not comfortable	4 (14.3)	6 (21)		
	N/A	0 (0.0)	1 (4)		

^a^N/A: not applicable.

## Discussion

### Principal Findings

Given EM’s high rates of burnout and the vulnerable role medical students hold as trainees, medical students undergoing their EM clerkship are at high risk of emotional strain and stress [15]. The rising focus on physician mental health extends to those in training [8]. There is a gap in understanding how prepared medical students feel in identifying and addressing their emotional response and the impact experiences in EM may have upon them. This study assessed medical students’ preparation to better understand their needs and guide interventions toward key priority areas of focus.

This study found that, regardless of whether students had already witnessed the care of a critically ill patient, most students did not feel prepared to identify or address the emotional impact associated with these situations. This highlights the need to train students early and normalize the emotional impact of working in medicine [19,20]. The stigma associated with mental health in health care has pervaded the classroom and hospitals, and to build structures to prevent burnout, we must begin early to help trainees identify feelings of anxiety, depression, or stress as they experience them [13,21-23]. A proactive approach would provide students with the tools and resources they need to adequately identify their emotions and connect to appropriate resources when needed.

It is essential for institutions not only to have resources available to support students’ well-being and mental health but also to make these resources readily accessible and easy to navigate. Our findings reveal that even in an environment where resources are present, students may be unaware of these resources or may not be comfortable accessing them. The University of Pennsylvania School of Medicine has a robust infrastructure within the medical school, a separate web-based mental health and well-being platform, accessible to the entire health system community [24]. However, students in this study remained unaware of their availability or accessibility. Institutions must work to incorporate these resources into the clinical and teaching environments to reduce the stigma that may prevent students from accessing them.

Finally, the significant differences in feelings of preparedness between those students who had and those who had not witnessed the care of critical care patients suggest that students do not feel prepared to identify the emotional impact such experience may have until it has happened. It is important to act proactively to prepare students to experience the care of critical patients. EM provides an ideal environment to do so, as the likelihood that students will be placed in an emotionally charged setting is high [17,25]. Venues such as critical care, pediatrics, obstetrics and gynecology, as well as surgery provide other opportune areas for schools to think about deploying focused interventions to where students may need them most.

### Limitations

This study has some limitations. First, to protect confidentiality and privacy, we did not collect demographic information. This prevented us from analyzing how these findings might differ by age, race, or ethnicity. Second, as medical students self-selected to participate, selection bias may play a role. The participants surveyed here may not accurately represent the experiences of all medical students in the clerkship program. Additionally, this study was performed at a single urban, academic program and may not be applicable to all medical students at other various institutions. We are also limited by a sample of 56 students, which may further limit our ability to generalize these findings.

### Conclusions

Similar to other roles in health care, medical students do not feel fully prepared to identify or address the emotional impact of working in acute care. Additionally, they lack awareness of or comfort with accessing institutional resources designed to support their well-being. Medical students who have not witnessed the care of a critically ill or injured patient were more likely to feel unprepared in identifying or addressing the emotional impact such an event might have on them. These findings can help inform and guide interventions by educational and academic leaders. The aim would be to create and promote training environments that empower students with tools to identify their own emotions and connect to well-being resources. We need to normalize the conversation around mental health in the health care workforce and reduce stigma early in medicine, beginning with our medical students.

## Abbreviations

EM: emergency medicine

## Data Availability

The data sets generated and analyzed during the study are not publicly available due to the sensitivity of data content in responses, but deidentified data are available by email request to the corresponding author upon reasonable request.
